# Community mental health in Europe: integrate science, practce and lived experience

**DOI:** 10.1192/j.eurpsy.2025.34

**Published:** 2025-08-26

**Authors:** R. Keet

**Affiliations:** FIT-academy, GGZ Noord-Holland-Noord, Heerhugowaard, Netherlands

## Abstract

**Introduction:**

The transition from institutional to community-based mental health care has been a key policy goal for decades. Despite progress, challenges such as inadequate funding, political prioritization, and a lack of clear frameworks persist. The European Community Mental Health Services Provider (EUCOMS) Network created a shared vision.

**Objectives:**

EUCOMS aims to outline the principles and key elements for implementing effective community-based mental health care systems. The primary goal is to bridge the gap between evidence, policy, and practice while promoting recovery-oriented, inclusive, and effective care models.

**Methods:**

The development process involved a collaborative, multi-phase approach. Starting with expert workshops and literature reviews, the framework was refined through input from 100 stakeholders, including mental health professionals, researchers, and peer experts across Europe. Structured feedback rounds and consultation workshops further shaped the consensus.

**Results:**

EUCOMS identifies six principles for high-quality community-based mental health care: **Human Rights:** Ensuring dignity, liberty, and equality for all individuals, with a focus on reducing stigma and discrimination. **Public Health:** Adopting a population-based approach to promote mental health and prevent illness. **Recovery:** Supporting personal, functional, and clinical recovery through individualized care. **Effectiveness:** Implementing evidence-based interventions and practices tailored to local contexts. **Community Networks:** Strengthening formal and informal support systems to enhance resilience. **Peer Expertise:** Integrating the lived experiences of service users into service design, delivery, and evaluation. In my presentation I will focus on how these principles are turned into practice in 3 countries in 3 different regions: The Netherlands, Portugal and Ukraine. The Netherlands: the nationwide implementation of the Flexible Assertive Community Treatment model, professionalization of peer expertise and the ecosystem approach on mental health. In Portugal, a nationwide guide for establishing and evaluating community mental health teams in Portugal, promoting integrated, accessible care. In Ukraine, the a nationwide All Ukraine mental Health program was launched during the war, implemented supported by all ministries supported by WHO.

**Conclusions:**

The EUCOMS Network presents a comprehensive framework for regional implementation of community-based mental health services. These principles advocate for systems that are inclusive, recovery-oriented, evidence-based, and integrated within the community. The model requires adaptable implementation, considering cultural, economic, and systemic contexts. Future efforts should focus on stakeholder engagement, capacity building, and sustained advocacy to align policy and practice with these principles.

**Disclosure of Interest:**

None Declared

